# Nitrogen Limitation of Pond Ecosystems on the Plains of Eastern Colorado

**DOI:** 10.1371/journal.pone.0095757

**Published:** 2014-05-13

**Authors:** John A. Mischler, Philip G. Taylor, Alan R. Townsend

**Affiliations:** Institute of Arctic and Alpine Research and Department of Ecology and Evolutionary Biology, University of Colorado, Boulder, Colorado, United States of America; Royal Netherlands Institute of Sea Research (NIOZ), Netherlands

## Abstract

Primary production in freshwater ecosystems is often limited by the availability of phosphorus (P), nitrogen (N), or a combination of both (NP co-limitation). While N fixation via heterocystous cyanobacteria can supply additional N, no comparable mechanism for P exists; hence P is commonly considered to be the predominant and ultimate limiting nutrient in freshwater ecosystems. However, N limitation can be maintained if P is supplied in stoichiometric excess of N (including N fixation). The main objective of this study was to examine patterns in nutrient limitation across a series of 21 vernal ponds in Eastern Colorado where high P fluxes are common. Across all ponds, water column dissolved inorganic N steadily decreased throughout the growth season due to biological demand while total dissolved P remained stable. The water column dissolved inorganic N to total dissolved P ratios suggested a transition from NP co-limitation to N limitation across the growth season. Periphyton and phytoplankton %C was strongly correlated with %N while %P was assimilated in excess of %N and %C in many ponds. Similarly, in nutrient addition bottle assays algae responded more strongly to N additions (11 out of 18 water bodies) than P additions (2 out of 18 water bodies) and responded most strongly when N and P were added in concert (12 out of 18 water bodies). Of the ponds that responded to nutrient addition, 92% exhibited some sort of N limitation while less than 8% were limited by P alone. Despite multiple lines of evidence for N limitation or NP co-limitation, N fixation rates were uniformly low across most ponds, most likely due to inhibition by water column nitrate. Within this set of 18 water bodies, N limitation or NP co-limitation is widespread due to the combination high anthropogenic P inputs and constrained N fixation rates.

## Introduction

Large-scale human alteration of nitrogen (N) [1], [2] and phosphorus (P) [3]–[5] cycles has substantially changed the absolute and relative supply rates of limiting nutrients in a broad variety of Earth’s ecosystems. Eutrophication of freshwater aquatic ecosystems [6]–[11] is one widespread consequence of such nutrient enrichment. Eutrophication can cause harmful algal blooms and bottom water hypoxia (dead zones) which in turn pose risks to fisheries resources, ecosystem services, and human health and recreation [12], [13]. Recent estimates for the United States suggest that cultural eutrophication has an annual cost of more than $2 billion per year [14]. As such, understanding the drivers and mechanisms of eutrophication has enormous societal and economic relevance [15], [16].

Phosphorus has long been identified as the ultimate limiting nutrient (and thus the causative agent of eutrophication when received in excess) within freshwater ecosystems [6], [17], [18], mainly because of strong observed relationships between total P and chlorophyll (an index of algal abundance) [19], [20]. Schindler [21] provided a mechanistic understanding of this relationship in a set of landmark multi-year whole lake nutrient enrichment experiments in which N derived from water column N fixation [8], [22], [23] and subsequent N regeneration and recycling from accumulated N in the sediment [24], [25] fully offset the N deficit induced by imbalanced experimental inputs of N and P over multiple growth seasons [26], [27]. This assertion that P is the eventual limiting nutrient in most freshwater ecosystems (the “P paradigm”) has focused efforts to avoid or reverse eutrophication solely on P control and removal (see [16] for review).

However there is accumulating evidence of N limitation [28]–[30] or NP co-limitation [31], [32] of primary production within a wide variety of freshwater ecosystems, prompting an active debate about the use of P-only control to avoid/reverse eutrophication across diverse aquatic ecosystems [15], [16], [25], [33]–[36]. N limitation or NP co-limitation can occur when N supply to the system is outpaced by P, (i.e. when rates of N fixation plus hydrologic supply are lower than the Redfield ratio of 16∶1 - the optimal stoichiometry for phytoplankton growth [37]). This dynamic can occur in aquatic systems that receive low N:P ratio hydrologic inputs from sewage, concentrated animal feeding operations (CAFOs) and/or fertilizer sources coupled with an inability of N fixation to ameliorate these N deficiencies on relevant timescales [15], [36].

The South Platte River Basin - which spans Colorado, Wyoming, and Nebraska - is one such region. The Basin contains a number of off-stream reservoirs, many with accompanying vernal ponds and wetlands. These water bodies serve as agricultural water storage, recreational areas, wildlife habitat, and aquifer recharge sites. Nutrient concentrations in the South Platte River are among the highest measured by the NAWQA Program [38] due to the high incidence of municipal wastewater treatment plants, CAFOs, and irrigated/fertilized cropland across eastern Colorado in conjunction with a low base flow [39]–[42]. These P-rich anthropogenic nutrient sources, along with high denitrification rates [43], explain the low DIN:TDP molar ratios (between 0.5 and 5.0) in the Basin’s reservoirs and wetlands [44], [45]. In this area, and others like it, the sustained supply of high concentration, low N:P nutrient inputs could be fundamentally shifting pond nutrient limitation from a pre-anthropogenic P limited regime to an N limited regime [46]. Even if anthropogenic sources of P were reduced, the P accumulated in the sediments could maintain N limitation for years or decades to come as sediments return large quantities of P back to the water column [47]–[51]. Imbalanced P-rich N:P supply rates have induced stable N limitation in some cases (e.g., [29], [30], [52]–[54]) and weakened the correlation between indices of primary productivity (such as chlorophyll a (chl a)) and P [55]–[57] thereby emphasizing the capacity of high P fluxes to force systems into N limitation, leaving N fluxes as the key determinate of eutrophication.

The goal of this study was to examine patterns in nutrient limitation across a series of vernal ponds filled with South Platte River water. Information on nutrient limitation in aquatic ecosystems can aid managers in maintaining ecosystem health and avoiding the harmful effects of cultural eutrophication. We combined field-based observational measurements of water chemistry and other relevant environmental data with a series of laboratory experiments to assess nutrient limitation of primary production. Given the long and well-documented history of P-rich inputs [44], we predicted that the pond ecosystems would be N limited, and thus, that at least for the near-term, any attempts at avoiding eutrophication would need to focus on decreasing N availability.

## Materials and Methods

### Site Description

All data were collected within two complexes of shallow (<3 m) vernal ponds located on the plains of Eastern Colorado. The Andrick Ponds State Wildlife Area and The Teal Hunting Lodge (40°22′16.77″ N, 104°06′24.89″ W; 13 ponds) comprise the western set of ponds while the Brush Prairie Ponds State Wildlife Area (40°12′46.68″ N, 103°38′37.53″ W; 8 ponds) is 40 km to the E-SE. All ponds are located on public land administered by the Colorado Division of Wildlife and all necessary permits were obtained for this study, which complied with all relevant regulations. Ponds at these sites form within shallow depressions in the uniformly sandy soil. They are replenished via irrigation ditches with water sourced from agricultural runoff and the South Platte River when water is available. All ponds at these sites often are filled to capacity in the late spring when irrigation water is abundant, but a lack of surplus water later in the season leads to gradual evaporation throughout the summer. Though some ponds may completely dry up at the end of the summer, others retain water (though at much lower levels). Because of the constant throughput of water in the spring, water residence times during the peak of the growth season may be short.

### Field Data Collection and Analytical Analyses

Field campaigns were conducted during the growth seasons (end of May to mid August) of 2011 and 2012 to investigate pond nutrient limitation. In 2011, the water column and periphyton were sampled across 21 ponds to assess nutrient limitation from a stoichiometric perspective. We followed up on observed field patterns by collecting water samples from all previously surveyed ponds which held water in July 2012 (17 of 21 ponds) and conducted nutrient addition bottle assays and nitrogen fixation experiments to empirically determine the potential for phytoplankton to respond to N, P, and N+P additions as well as their potential to fix atmospheric nitrogen. We also measured seston C, N, and P in these samples. Sampling was limited by the ephemeral nature of the ponds as well as logistical considerations of pond management by the Colorado Division of Wildlife for waterfowl hunting.

During the 2011 growth season surface water samples were collected in triplicate weekly from each of the 21 ponds. Water was filtered with a GF/F (Whatman glass fiber filter, nominal pore size) into acid-washed polypropylene containers and frozen for later analyses. All surface water samples were analyzed for total dissolved organic carbon (TDOC), total dissolved nitrogen (TDN), soluble reactive phosphorus (SRP), NH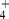, and NO
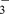
+NO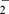. TDOC and TDN were determined in all samples using a high temperature combustion TDOC/TDN analyzer (Shimadzu TOCvcpn, Kyoto, Japan). NO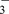 and SRP were analyzed colorimetrically on an Alpkem autoanalyzer (OI Analytical, College Station, TX, USA) using the cadmium reduction [58] and the ammonium molybdate ascorbic acid methods [59] respectively. NH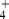 was analyzed colorimetrically on a BioTek Synergy 2 Multi-Detection Microplate Reader (BioTek, Winooski, VT, USA). Total dissolved phosphorus (TDP) in water samples was determined by a potassium persulfate/sodium hydroxide digestion to convert organic phosphorus to SRP and measured as above. Conductivity, pH, temperature, and Secchi depth were measured in each pond (Table S1). In addition, a 6 liter composite water sample was collected from each pond on July 16, 2012 and prefiltered through a 153 *μ*m Nitex screen. Four liters were used for the nutrient addition bottle assay experiment, 0.5 liters for the nitrogen fixation syringe experiment, and 1.5 liters for a composite seston sample.

Periphyton samples were also collected from all 21 ponds surveyed in 2011. Periphyton was collected by selecting movable substrate (wood, dead reeds, aquatic macrophytes) every ∼30 m around the entire circumference of the pond and placing these substrates with included periphyton into plastic bags and transporting them on ice to the lab. For ponds with circumferences larger than 300 m, 10 equally spaced samples were taken around the circumference. Once in the lab substrate samples were mixed with DI water and rigorously shaken for 30 seconds to force as much periphyton as possible into suspension. This periphyton slurry was then filtered with a vacuum pump onto a Whatman GF/F filter. Seston samples were obtained from prefiltered (153 *μ*m Nitex screen) water samples by filtering with a vacuum pump onto a Whatman GF/F filter. The seston and periphyton covered filters were dried at 60°C for 48 hours and placed in a freezer until analyzed for element composition.

Four equally-sized punches from the dried seston or periphyton covered filters were massed and packed in tins for C and N analyses. The average mass of 4 punches of a clean GF/F filter was subtracted from the dried seston or periphyton covered filter punches to determine the actual mass of seston or periphyton alone. Filter blanks were also prepared in the same was as the samples. Percentage of carbon (%C) and nitrogen (%N) was determined using a Carlo Erba EA 1110 elemental analyzer (CE Elantech, Lakewood, New Jersey, USA). Phosphorus (P) extraction was performed after [60], [61]. Four seston or periphyton punches were placed in 30 ml glass vials and ashed at 500°C. P was extracted using 5 ml of 1 N HCl heated to 80°C for 30 minutes and then diluted with 5 ml of DI water. P was determined in the diluted leachate using the colorimetric analysis above. All GF/F filters were heated to 500°C for 4 hours and rinsed with DI water to eliminate contamination and ensure nutrient-free conditions before use. Ten GF/F filter blanks were randomly selected for nutrient analyses. Nutrient levels were measured as described above, with all samples yielding nutrient levels below detection limits for %C, %N, and %P.

On July 16, 2012 surface water samples were collected from 17 ponds surveyed in 2011 (4 were completely dry) for chemical analyses listed above as well as for laboratory experimental analyses. An additional 6 L composite water sample was collected from each pond and prefiltered through a 153 *μ*m Nitex screen. Four liters were used for the nutrient addition bottle assay experiment and 0.5 liters for the nitrogen fixation syringe experiment, which are both described below.

### N and P Enrichment Experiment

A nutrient-enrichment bioassay experiment was performed in July 2012 using water from 17 of the 21 ponds surveyed in 2011 as well as an irrigation ditch supplying water to some of the ponds. Six liters of water was composited into a single sample from each pond on July 16, 2012 and prefiltered through a 153 *μ*m Nitex mesh, with aliquots partitioned for measurement of water column N fixation. Our bioassays were modeled after the US Environmental Protection Agency protocol for algal assay bottle tests using natural assemblages of phytoplankton [28], [62], [63].

Treatments consisted of 4 repetitions each of (i) a control (no nutrient added), (ii) +N, (iii) +P, and (iv) +N+P. Nutrient treatments were 320 *μ*mol/L N (i.e., 4480 *μ*mol/L of NH_4_NO_3_) and 20 *μ*mol/L P (i.e., 620 *μ*mol/L of KH_2_PO_4_) in the single nutrient addition treatments and a combination of both in the +N+P treatment. The nutrient ammendments were adjusted to overcome the high P levels in the pond water and to achieve a Redfield N:P ratio of 16∶1. All bottles (240 mL) were incubated in the lab at a constant temperature (25 to 26°C) for 5.5 days under a 16 h light:8 h dark cycle using natural spectrum grow lights (20–27 *μ*mol/m^2^/s, Instant Sun natural spectrum fluorescent tube light, 2100 photopic lumens, 6280°K color temperature, 94.5 color rendering index). The bottles were shaken twice daily and randomized once daily. At the end of the experiment all water within each bottle (200 ml) was filtered onto GF/F filters and each filter was folded, placed in foil, and frozen for chlorophyll analysis (corrected for pheopigments via hydrochloric acid additions) [64], [65]. Chl a was determined using a FluoroMax-2 200–900 nm spectrofluorometer (HORIBA Scientific Edison, NJ).

### Water Column N Fixation

Nitrogen fixation rates were estimated by the acetylene reduction method [66] in water collected in 2012 from each of the 17 ponds and the irrigation ditch. Sixty ml polypropylene syringes were used as assay vessels after blank tests showed no leakage or in-situ ethylene production over our assay time period (3 to 4 hours) [67]. Four syringes were used for each pond (3 replicates, 1 control) after the methods of [45], [68]. An integrated water sample from each pond served as the sample water from which subsamples were drawn into each syringe. Each syringe was rinsed with sample water and 40 ml of sample water was drawn into the syringe, air was purged, and water volume in each syringe was adjusted to ∼30 ml. Five ml of acetylene (generated via hydrolysis of calcium carbide in DI water and stored in a bladder) was added to each replicate syringe, which was sealed using a valve and moderately agitated for 10 seconds. Five ml of air were added to each control syringe which were sealed and agitated in the same manner as those that received acetylene. Blanks were prepared using sterile DI water instead of sample water to account for background ethylene in the acetylene source.

The syringes were incubated for 3 to 4 hours at a constant temperature (25 to 26°C) under natural spectrum fluorescent grow lights (30–40 *μ*mol/m^2^/s). These temperature and light conditions are common in the epilimnia (upper part of the water column) of many lakes [69]–[71]. At the end of the incubation, 20 ml of air was drawn into each syringe and each syringe was shaken vigorously for 30 seconds to equilibrate the liquid and vapor phases. Aqueous and vapor volumes were recorded following equilibration to account for partitioning of ethylene between aqueous and vapor phases [66], [72]. The incubation was halted by removing a sample of the headspace and placing it in a 5 ml vacutainer (Becton, Dickinson and Company, Franklin Lakes, NJ, U.S.A.) that had been previously manually evacuated to ensure that no other substances were present within the vacutainer. Ethylene was measured using a Shimadzu 14-A Gas Chromatograph equipped with a flame ionization detector (330°C) and a Poropak N column (110°C; Supelco, Bellefonte, Pennsylvania, USA) at an oven temperature of 80°C. For each sample a 3 ml aliquot of gas was removed from the 5 ml vacutainer using an airtight glass syringe fitted with a valve that was closed prior to and following vacutainer sampling. This subsample was injected into the instrument by opening the valve and forcing all gas out of the syringe. Ethylene concentration was determined by comparing to a standard curve containing known ethylene concentrations. After accounting for variables affecting ethylene recovery (temperature and relative volume of headspace; [73]), ethylene production was converted to nitrogen fixation with a 4∶1 ethylene/dinitrogen conversion ratio [67].

### Statistical Analyses

Water column nutrient data were compared between the ditch and an aggregation of all pond data using t-tests. Correlations were examined using the Pearson product-moment correlation coefficient (r). N fixation rates were compared to DIN:TDP ratios using the MannWhitney U test. Where appropriate, values are displayed as means with ±1 SE. Nutrient limitation was assessed using the ratio of DIN:TDP as measured in the water column during the 2011 growth season using the thresholds of Morris et al. [31]. According to [31] a water body is likely to be P limited if its DIN:TDP molar ratio is above ∼18 and N limited below ∼2.2. Co-limitation is thought to predominate in ponds with DIN:TDP molar ratios between ∼2.2 and ∼18. These thresholds were chosen specifically because of their widely accepted use and this study’s explicit testing of the efficacy of different nutrient ratios in predicting nutrient limitation (though other thresholds exist - [74], [75]). The DIN:TDP ratio is a measure of nutrient supply to phytoplankton and has been demonstrated to be accurate 80% to 90% of the time in predicting limiting nutrients compared with results from bioassay experiments [31]. For N, the largest bioavailable pool tends to be DIN while P includes both SRP and dissolved organic phosphorus (DOP). DOP is bioavailable to phytoplankton because of the excretion of alkaline and acid phosphatases that enzymatically cleave phosphate groups off organic molecules [76]. The ambient DIN threshold above which water column N fixation becomes unfavorable (20 *μ*g/L) was taken from work in adjacent pond systems by [45].

Periphyton and seston C:N, N:P, and C:P molar ratios were compared to modified Redfield ratios from [77]. Because Redfield ratios are empirically developed stoichiometric ratios from deep ocean phytoplankton, slightly different ratios are expected for freshwater periphyton because of differences in physiology and life strategies. Hillebrand et al. [77] developed empirical stoichiometric ratios for optimal freshwater periphyton growth under balanced N:P supply rates (C:N:P = 119∶17:1) which are stable against changes in abiotic conditions. Because optimal growth takes place at these modified Redfield ratios and periphyton’s capacity to store P is enhanced when excess P is present in the environment (luxury P uptake - [78]) P accumulated in excess of C or N is indicative of excess P supply. The goodness of fit of periphyton/seston with the modified Redfield ratios of [77] were evaluated using coefficients of determination (R^2^).

Results for the nutrient addition (+N, +P, +NP) bottle assays were compared qualitatively across treatments to determine broad-scale patterns in nutrient addition. A statistical approach, using a two-way ANOVA and post hoc pairwise comparisons following [79] was used to distinguish between various types of nutrient limitation. Single nutrient limitation was indicated by a significant chl a response to only one nutrient (N or P alone) addition in the 2-way ANOVA with no significant N-P interaction while an additive dual nutrient limitation was indicated by a significant chl a response to both N and P addition alone in the 2-way ANOVA with no significant N-P interaction. Sequential N co-limitation was indicated by a significant interaction in the chl a response to interaction in the 2-way ANOVA and post hoc pairwise comparisons where ChlNP > ChlN > ChlC = ChlP while sequential P co-limitation was indicated by a significant interaction in the chl a response to interaction in the 2-way ANOVA and post hoc pairwise comparisons where ChlNP > ChlP > ChlC = ChlN. Strict co-limitation with N and P was indicated by a significant interaction in the in 2-way ANOVA and a post hoc pairwise comparison indicating ChlNP as the only significant difference from the control. No significant terms in the 2-way ANOVA indicated a lack of response to nutrient enrichment. N fixation rates were computed as *μ*mol per L per day assuming 10 hours of active fixation per day and uniform fixation with depth; rates were extrapolated to an annual flux assuming a 65 day growth season [8].

## Results

### Biogeochemical Patterns in N and P

**Figure 1:**
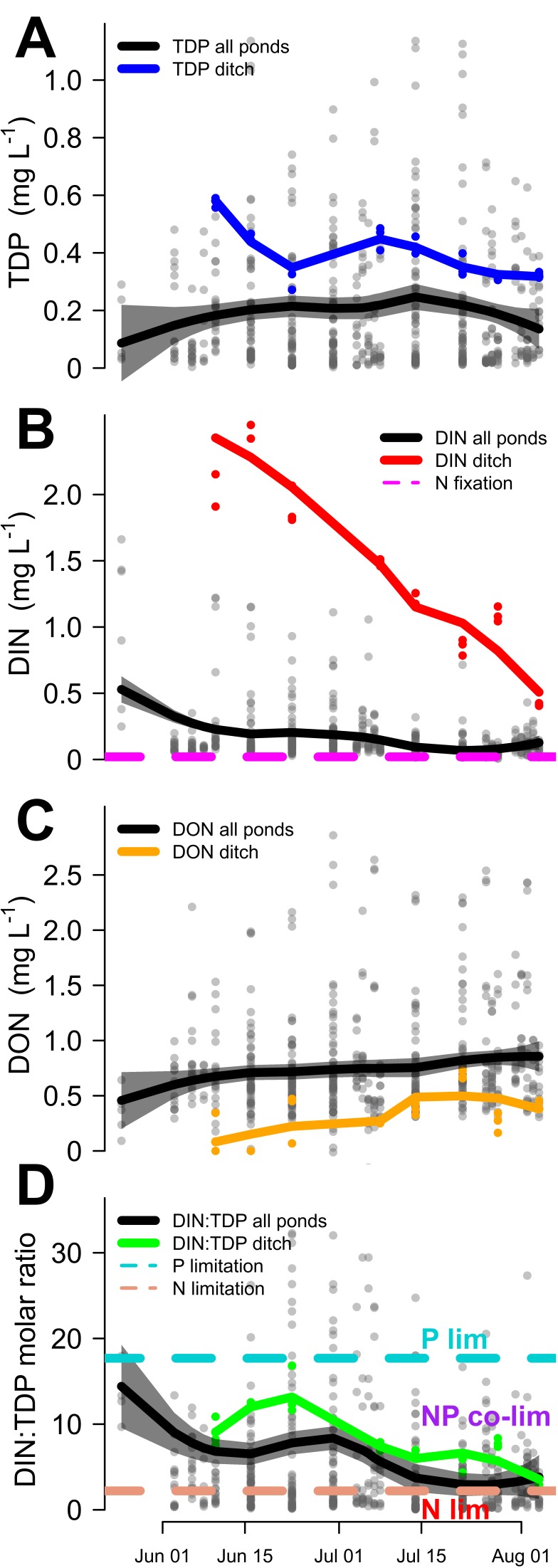
Weekly water column biogeochemical measurements from 21 ponds indicate nitrogen limitation. Water samples taken weekly throughout the 2011 growth season (late May to early August) from 21 ponds and an irrigation ditch providing water to the ponds. (A) TDP concentrations of the pond water (black) and the supply ditch (blue). (B) DIN concentrations (NO
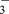
+NO
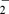
+NH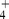) of the pond water (black) and the supply ditch (red) compared to the threshold (magenta) from Bradburn et al. [45] above which DIN concentrations inhibit water column N fixation. (C) DON concentrations of the pond water (black) and the supply ditch (orange). (D) DIN:TDP molar ratios of the pond water (black) and the supply ditch (green) compared to the thresholds of Morris et al. [31] for P-limitation (above cyan line), NP co-limitation (below cyan line and above red line), and N-only limitation (below red line). Gray confidence intervals around the pond data are ±2 SE.

Across the growth season the ditch had higher concentrations of all N and P species compared to the ponds except for DON (Figure 1A–D, TDP = 0.40±0.02, 0.20±0.01, p = 1.7e-11; DIP = 0.23±0.02, 0.15±0.007, p = 0.003; DIN = 1.41±0.15, 0.15±0.008, p = 4.4e-09; DON = 0.31±0.04, 0.76±0.02, p = 1.0e-10). The mean DIN in the ditch was almost 10 times higher than the mean DIN in the ponds (Figure 1B) while the mean TDP was two times as high (Figure 1A). Consequently, the mean DIN:TDP ratio of the ponds was significantly (p = 0.003) lower than that of the ditch (Figure 1D). TDP concentrations in most ponds showed little variation over time (0.20±0.01), which was half the mean ditch concentration (0.40±0.02) (Figure 1A). In contrast, DIN concentrations in both the ditch and the ponds steadily declined throughout the growth season, reaching a low at the end of the summer (Figure 1B). DON concentrations were much higher than DIN, varied little (0.76±0.02), and were twice as high as the ditch mean (0.31±0.04, Figure 1C).

### Indicators of Nutrient Limitation

#### Water column stoichiometry

**Figure 2:**
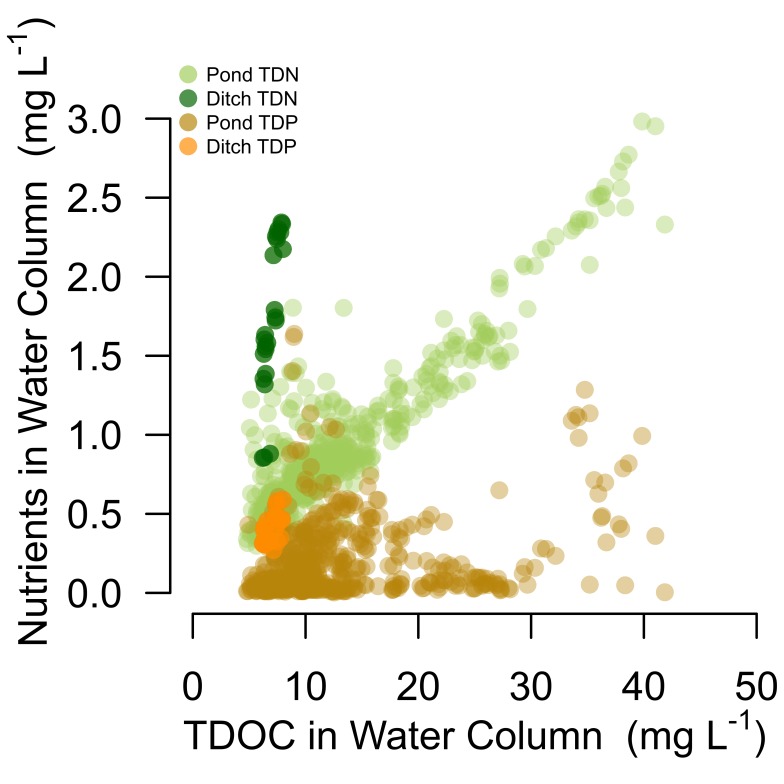
Weekly water column measurements of TDOC vs. TDN and TDP from 21 ponds indicate N limitation. Water samples taken weekly throughout the 2011 growth season (late May to early August) from 21 ponds (lighter points) and an irrigation ditch providing water to the ponds (darker points). TDN and TDP concentrations are higher in the ditch water than the pond water when compared to TDOC. There is a strong positive correlation between TDN and TDOC (r = 0.92, p < 2.2e-16) and a much weaker relationship between TDP and TDOC (r = 0.27, p = 8.0e-11) indicating N as limiting C fixation in the system. Alternatively the lack of a stong correlation between TDP and TDOC could arise from luxury P uptake decoupled from C fixation.

DIN:TDP ratios of ditch water were within the thresholds of N and P co-limitation throughout the growth season (Figure 1D, [31]). In most ponds the DIN:TDP ratios strongly decreased throughout the season, driven by a decline in DIN concentrations. From a stoichiometric perspective, the decline in DIN:TDP values indicated a shift from N and P co-limitation to N limitation by the end of the growth season (Figure 1D). Water column TDOC was closely correlated with water column TDN (r = 0.92, p < 2.2e-16; Figure 2), while water column TDP exhibited a much weaker relationship with TDOC (r = 0.27, p = 8.0e-11; Figure 2).TDOC did not exhibit a strong trend across the season (Figure S2).

#### Periphyton and seston stoichiometry

**Figure 3:**
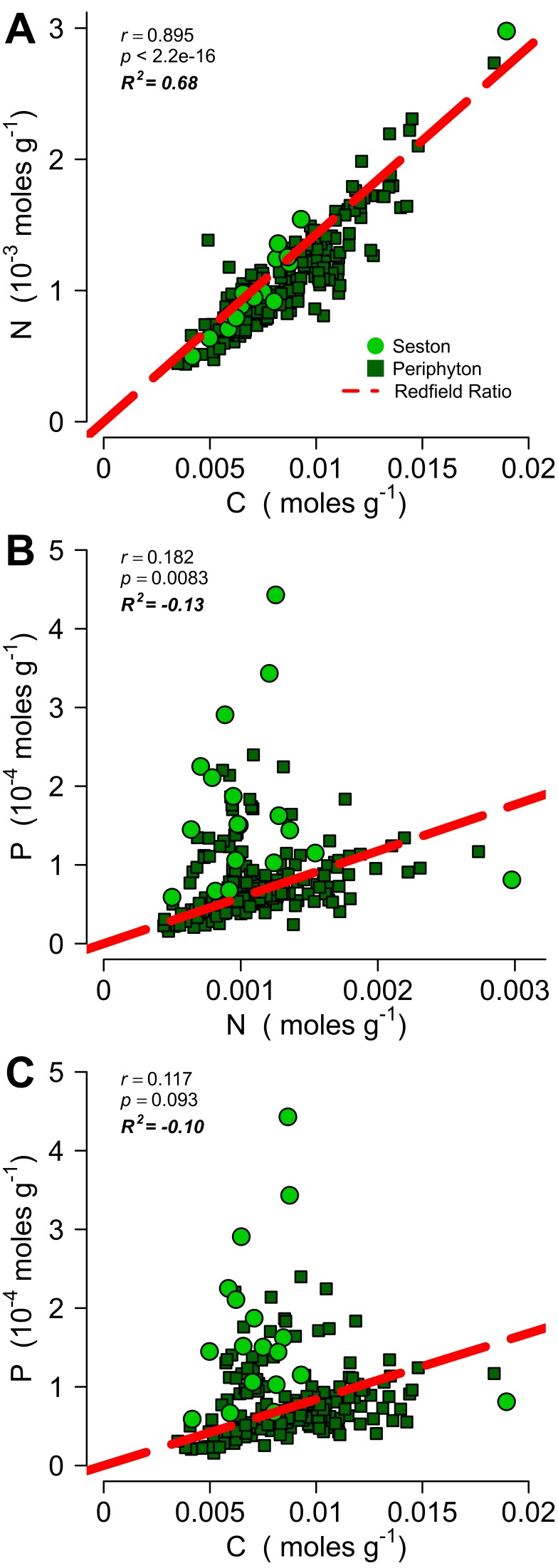
Periphyton and seston stoichiometry within ponds. Periphyton (dark green squares sampled July 2011) and seston (light green circles sampled July 2012) molar nutrient contents compared to the modified Redfield ratio (C:N:P = 119∶17:1) for periphyton [77]. (A) C content is tightly coupled to N content (r = 0.89, p < 2.2e-16) and follows the modified Redfield ratio (R^2^ = 0.68) indicating a strong dependence of C accumulation on N content. (B) P content is in excess of N content for many samples producing a much weaker correlation (r = 0.18, p = 0.008) and a poor fit to the modified Redfield ratio (R^2^ = −0.13) and (C) P content is in excess of C content for many samples with no significant correlation and a poor fit to the modified Redfield ratio (R^2^ = −0.10); both indicating luxury P uptake and a lack of dependence of C accumulation on P content.

Periphyton stoichiometry and seston stoichiometry were measured during July 2011 and July 2012 respectively (Figure 3A–C). Organic C and N concentrations were tightly linked for both periphyton and seston (r = 0.89, p < 2.2e-16; Figure 3A). Periphyton/seston P had a much weaker relationship with N (r = 0.18, p = 0.008) and no significant relationship with organic C (r = 0.12, p = 0.09). The modified Redfield C:N ratio [77] was a good fit for field-derived periphyton/seston C:N ratios (R^2^ = 0.68; figure 3A), whereas field-derived N:P and C:P ratios were better fitted by their own means rather than the modified Redfield N:P and C:P ratios, emphasizing the modified Redfield ratios’ poor fit with P-related field-derived ratios at this site (R^2^ = -0.13, -0.10; Figures 3B, 3C). Seston and periphyton both commonly exhibited excess accumulation of P versus N (Figure 3B) and P versus C (Figure 3C) compared to the modified Redfield Ratio [37], [77] while rarely exhibiting excess N (Figure 3B) and excess C (Figure 3C) accumulation versus P.

#### Nutrient addition bioassay

**Figure 4:**
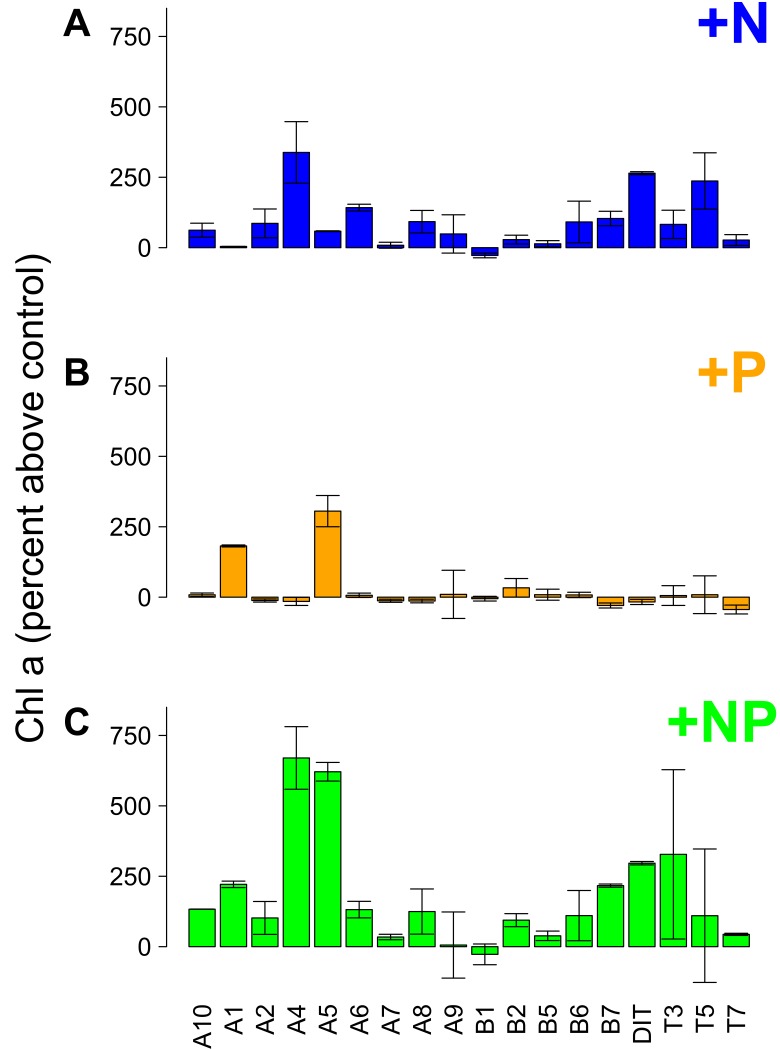
Results from bottle nutrient addition experiments. Percent change in chlorophyll a (chl a) after addition of (A) +N alone, (B) +P alone, and (C) +NP together, compared to controls (no nutrients added) from water taken from 17 ponds and the supply ditch in 2012. Chl a increased more than 50% above controls in: (A) 11 water bodies when N alone is added, (B) 2 water bodies when P alone is added, and (C) 13 water bodies when N and P are added together. While adding N alone induced chl a responses in most ponds, adding N and P in concert and the most consistent and largest magnitude effect.

**Figure 5:**
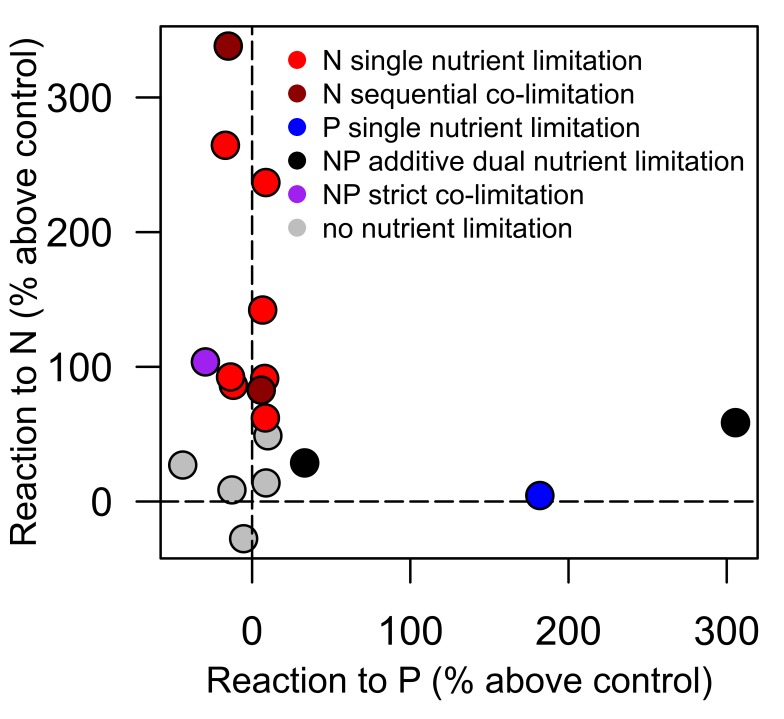
Nature of nutrient limitation observed in the nutrient addition bottle experiments. Percent change in chl a after addition of +N alone (y axis) and +P alone (x axis) compared to controls (no nutrients added) from water taken from 17 ponds and the supply ditch in 2012. Clustering along the y axis is indicative of N limitation while clustering on the x axis indicates P limitation. A two-way ANOVA was used to determine the type of nutrient limitation (colors) using the +N, +P, and +NP data after Elser et al. [79]. 12 of the 18 water bodies were limited by N alone or in combination with P, while only one pond exhibited P-only limitation. 5 ponds did not show a significant response to nutrient addition at the p < 0.05 level.

N-only nutrient additions produced positive chl a responses (50% above control values) in 11 water bodies (Figure 4A) while P-only additions produced chl a responses in only 2 ponds (Figure 4B). When N and P were added in concert 13 ponds showed a positive response to nutrient enrichment (Figure 4C). Of the 18 water bodies tested in the 2012 bioassay (Figure 5), 7 ponds showed single nutrient limitation by N while only one showed single nutrient limitation by P. One pond showed additive dual nutrient limitation to N and P indicating that production would increase by adding either N or P alone or in concert. Two ponds showed sequential N co-limitation indicating that chl a showed a response to N addition and an even greater response to NP addition but did not respond to P addition alone. In sequential N co-limitation P is only effective if N is added in concert but N is effective alone or in concert with P. One pond showed strict co-limitation with N and P. Of the bottles that responded to nutrient addition, all N only, N sequential, and NP co-limitations had DIN:TDP molar ratios less than 4.5, whereas P only and NP dual limitation occurred at DIN:TDP molar ratios greater than 4.5, indicating a higher threshold for shifting from N limitation to NP co-limitation than seen in [31]. N only, N sequential, and NP co-limitation was associated with higher TDP values (greater than 0.025 mg/L) whereas P only and NP dual limitation occurred at TDP < 0.025 mg/L. P only limitation occurred in only one pond (A1), which had a mean DIN:TDP molar ratio of 14.6.

### N Fixation

**Figure 6:**
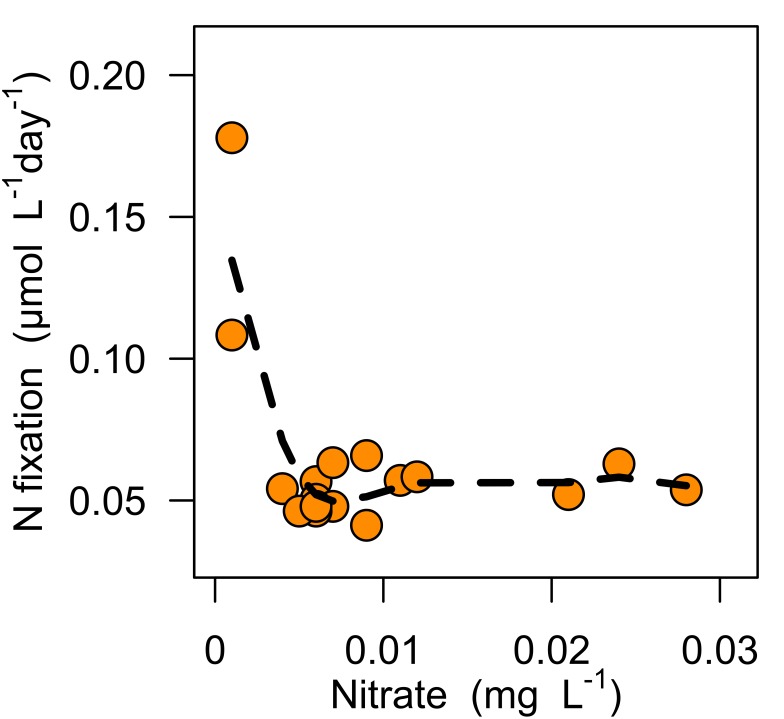
Nitrate concentration threshold for loss of competitive advantage of water column nitrogen fixation. Water Column N fixation rates were measured for water samples collected in July 2012. N fixation was not competitively advantageous when nitrate concentrations were higher than 4 *μ*g/L. This result is broadly consistent with the results of Bradburn et al. [45] from Jackson Reservoir (an adjacent water body). The N fixation data from B5 were not included in this plot due to it being anomalously high compared to all other ponds.

In general, N fixation rates were consistently low across all ponds (Figure 6) except for pond B5 which had an anomously high (8.9±0.005 *μ*mol-N/L/day) N fixation rate compared to the other samples. N fixation rates in all other ponds were lower than many other lab-derived values under similar light and stoichiometry levels [80], but are in line with estimates of N fixation from adjacent water bodies [45]. Similarly, [8] found a mean N fixation rate of ∼0.52 *μ*mol/L/day (range = 0.007 to 2.86 *μ*mol/L/day) when looking across 20 freshwater field studies. If the observed N fixation rate of 0.065 *μ*mol/L/day is extrapolated to a 65 day growth season N fixation contributes 0.06 mg of N per L per growth season, which is 12% of the mean standing stock of TDN in the 18 water bodies in this study (0.77 mg/L). There was no correlation between water column DIN:TDP and N fixation rates (p = 0.7), although N fixation rates were higher on average (0.14±0.03, 0.06±0.01; p<0.05) in ponds that had less than 4 *μ*g/L NO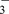 (Figure 6). Water nutrient concentrations, N fixation rates, and nutrient limitation statuses are listed in Table S2.

## Discussion

Observational and experimental results indicate that N limitation is common in the 18 water bodies in this study, with P being supplied in stoichiometric excess of N compared to biological demand [37], [77]. While P limitation is widespread across many freshwater bodies [16], our results confirm the importance of strict or partial N limitation in water bodies in the South Platte River basin as suggested by others [44], [45], [52]. We can infer the existence of persistent strict or partial N limitation throughout the growth season at the study sites based on water nutrient stoichiometry (Figure 1A–D, Figure 2). This inference is confirmed by measurements of seston and periphyton stoichiometry (Figure 3A–C), estimates of water column N fixation rates, and the results of nutrient addition bottle assays (Figures 4 and 5). On the plains of Eastern Colorado, P inputs appear to exceed both anthropogenic and *in situ* mechanisms for maintaining stoichiometric equilibrium, in ways that may have shifted many systems into N limitation.

### Evidence for N limitation

#### Field observations

Patterns in water column N and P chemistry suggest that soluble P is consistently in excess of autotrophic demand, suggesting a predominance of N limitation from a stoichiometric perspective (Figure 1D). The high P loads originate from the irrigation water, which is the predominant source of water and nutrients to the ponds in this study (Figure 1A–D). Groundwater is unlikely to play a role in nutrient supply as the area of study does not contain any N or P-rich lithologies [81] and groundwater plays a minor role within this network of irrigation ponds. The ditches deliver large amounts of DIN and TDP during the beginning of the growth season (Figures 1A–B), but large TDP fluxes occur throughout the growth season (mean = 0.40±0.02 mg/L, Figure 1A) without much variation whereas DIN supply steadily decreases from 2.32 mg/L at the beginning of June to 0.42 mg/L in August (Figure 1B). As a result, the ditch water DIN:TDP decreases from 12±0.9 to 3±0.1 moving from NP co-limitation towards strict N limitation [31] (Figure 1D).

On average, ponds contain lower concentrations of DIN and TDP than the ditch indicating that they are nutrient sinks. However, while pond DIN never exceeds ditch DIN (Figure 1B), many ponds contain TDP concentrations higher than ditch TDP concentration for at least part of the season (Figure 1A). As with other indicators, these data suggest that while available N is quickly used in these systems and converted to organic N (Figure 1C), available P remains in the water column because it is supplied in excess of biological demand. Almost 85% of the TDN in these study ponds is DON, compared to only about 25% of TDP being DOP, and the tight positive correlation between water column TDOC and TDN (Figure 2) highlights the reliance of C fixation on N supply. The relationship between TDP and TDOC is much weaker, although we note that the tendency for luxury uptake is much higher for P than N [82].

Similarly, patterns in seston and periphyton stoichiometry also suggest widespread N limitation in these 18 water bodies (Figure 3). Periphyton and seston accumulate P in excess of the modified N:P and C:P Redfield ratios (Figure 3C, 3D). While this finding indicates excess available P in the water column and is suggestive of N limitation, it is not direct evidence of the latter due to the inherent flexibility of P assimilation in autotrophs [78]. However, the tight relationship between periphyton/seston C and N and the close agreement with the modified C:N Redfield ratio indicates that N availability controls C fixation specifically and hence primary production within the benthos in general (Figure 3A). Periphyton in the study ponds tends to be more efficient than phytoplankton at ameliorating N deficiency to achieve stoichiometries closer to the modified Redfield (Figure 3), possibly because of periphyton’s faster and/or less constrained N fixation rates [83]–[86] or its ability to outcompete phytoplankton for available N in small ponds [87].

#### Experimental evidence

The results of the nutrient enrichment bioassay also support N limitation or NP co-limitation in many of the ponds in this study (Figure 5). The threshold ratios of Morris and Lewis [31] indicate P-only limitation at DIN:TDP molar ratios above 18, N-only limitation at molar ratios below 2, and NP co-limitation between 18 and 2 (Figure 1D). Our results were broadly consistent with these thresholds showing P-only limitation at DIN:TDP ratios above 15, N-only limitation at ratios below 4, and NP co-limitation between 15 and 4. Of the 13 ponds that exhibited nutrient limitation, 12 involved some form of N limitation (Figure 5). We conducted nutrient addition bottle bioassays to confirm nutrient limitation suggested by water column stoichiometry measured throughout the season. The utility of bottle-style assay results alone can be limited due to the omission of crucial ecosystem processes (large free-ranging organisms, periphyton nutrient cycling, sediment/water interactions, etc.) [88]. The results of our nutrient addition bottle assays were broadly consistent with our measures of water column, periphyton, and seston stoichiometry and reflect likely N limitation or NP co-limitation in the water columns of these study ponds. However, whole-pond nutrient addition experiments to capture the full ecosystem complexity would be advisable to inform large-scale management actions in these ponds.

### Why is N Limitation Prevalent?

The water column data (Figure 1), along with periphyton and seston stoichiometry (Figure 3) and nutrient addition bioassays (Figures 4 and 5) all suggest that N limitation both by itself and concurrently or reciprocally with P is widespread in this system of ponds. Such N limitation is most likely maintained by consistently imbalanced inputs of P versus N nutrient sources (Figure 1), as illustrated by the excess available P in the water column in conjunction with low DIN. Temporal differences in N and P supply (Figure 1) probably occur because (1) the main source of DIN (fertilizer runoff) is maximized at the beginning of the growth season while more of the P-rich nutrient sources (sewage, manure) occur steadily throughout the year and (2) the reservoirs that supply the ditches concentrate P and sequester/volatilize N [44]. Under such P-rich, N-poor conditions, N fixation by cyanobacteria has been reported to make up N deficits [8], [22], [23], especially as N fixing cyanobacteria are expected to dominate when water column N:P is low [21], [25], [27] and TDP is abundant. However recent work suggests this may not be the case [36], [89], [90].

N fixation often cannot keep pace with regular and consistent nutrient inputs containing large amounts of P and proportionally small amounts of N [21], [29], [30], [52]–[54]. Also, in our ponds N fixation appeared to be inhibited even at low NO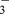 levels (4 *μ*g/L or higher, Figure 6). In nearby bodies Bradburn et al. [45] found that water column N fixation was inhibited at DIN concentrations above 20 *μ*g/L and Holl et al. [91] found similar inhibitory thresholds with NO_3_^−^ in marine cyanobacteria. Other factors such as turbulence, stratification, light availability, and minor elements (Fe and other trace metals) may also be important to N fixation rates in these systems. While DIN concentrations were uniformly low in the ponds in this study, all ponds maintained DIN concentrations above 20 *μ*g/L DIN (Figure 1B and Figure S1). In combination with low N fixation rates, low N:P ratios could be exacerbated by high levels of denitrification of NO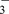 to N_2_ gas due to warm water temperatures (Table S1) [46], [92], [93] and high TDOC (Figure S2), which would lower bioavailable N. Mean annual rates of denitrification in the South Platte river below Denver, Colorado, are extremely high (0.51.62 g N m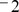 d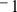 [94], [95]), five or more times higher than denitrification rates documented for many other US rivers [96]–[98]. If N losses due to denitrification outpace N inputs to aquatic systems, N limitation can be perpetuated if P remains available to support regenerated and new production [99]–[101].

There is no analogous removal pathway for P. Instead, excess P can accumulate in both organic (i.e. autotrophic luxury uptake, Figures 3B and 3C) and inorganic reservoirs (i.e. sorption to sediments), and regenerate as an internal source of P. TDP periodically exceeded TDP supplied from the ditch (Figure 1A), suggesting concentration of P within the ponds, perhaps via evaporation [102] or sediment sources [103]. Additionally, periphyton and seston accumulated P in excess of N (Figure 3B), thus preventing P burial during the growth season and reinforcing high rates of internal P regeneration. Altogether, our results suggest that these pond systems will maintain relative N deficiency because of constrained N fixation rates (Figure 6) combined with high potential for denitrification, high hydrologic P relative to N loads, and a longer-term capacity for sediment P regeneration.

Broadly, our study suggests that widespread increases in anthropogenic P loading can cause freshwater systems to receive nutrient inputs that are stoichiometrically enriched in P relative to N (such as the ponds in this study), and thus push aquatic ecosystems into at least proximate N limitation. In theory, this N limitation is reversible, but regeneration of P stored in sediments can prevent N and P balance over multi-year timescales [47]–[51], even after P loads loads have been decreased.

The bulk of policy instruments and management strategies aimed at decreasing freshwater eutrophication in the U.S. and elsewhere focus on P management [104]. However, a number of recent studies [15], [33]–[36] have highlighted the potential need for N control. In our study system, data suggest that remediation of N inputs would decrease eutrophication in the near-term. Substantial, long-term P control might eventually switch these systems into a P-limited state, but the legacy of past P inputs is likely to last for decades [47]–[49], meaning that any meaningful decrease of cultural eutrophication is almost certain to require a dual-nutrient strategy. Finally, we note that even where P-only control does successfully decrease eutrophication by inducing P limitation in previously N limited systems [105], [106], it can lead to higher water-column concentrations of reactive N species with consequent downstream impacts [107].

## Supporting Information

Figure S1 **Water column dissolved inorganic nitrogen (DIN) values per pond compared to the experimental N fixation threshold determined by Bradburn et al.** [45] **.** Each box contains per pond DIN values across the growth season. The red dashed line is the DIN threshold (20 *μ*g/L) above which water column N fixation rates decrease substantially. This threshold was determined by field experiments by Bradburn et al. [45] using adjacent water bodies. While DIN concentrations were low in many of the study ponds, DIN levels were consistently maintained above this 20 *μ*g/L threshold suggesting a water column N control on N fixation within the study ponds.
(TIFF)

Figure S2 **Total dissolved organic carbon (TDOC) concentrations for all ponds across the growth season.** TDOC measurements across the growth season remained steady (mean = 13±0.3 mg/L) with no systematic changes across the growth season. The ponds maintained TDOC concentrations above those of the ditch indicating the ditch is not a significant source of TDOC to the ponds.
(TIFF)

Table S1 **Conductivity, pH, temperature, and dissolved oxygen measurements throughout the 2011 growing season.** These are measurements taken in triplicate within ponds across the 2011 growing season: Conductivity (C1,C2,C3), pH (pH1,pH2,pH3), Temperature (T1,T2,T3), and Dissolved Oxygen (DO1,DO2,DO3).
(PDF)

Table S2 **Water Chemistry and Seston Data for Waters Used in 2012 Experiments.**
(PDF)
