# Supplementary figures and images for: Nitrogen Limitation of Pond Ecosystems on the Plains of Eastern Colorado
Source: PLoS One. 2014 May 13;9(5):e95757. doi: 10.1371/journal.pone.0095757 (PMC4019484; doi:10.1371/journal.pone.0095757)

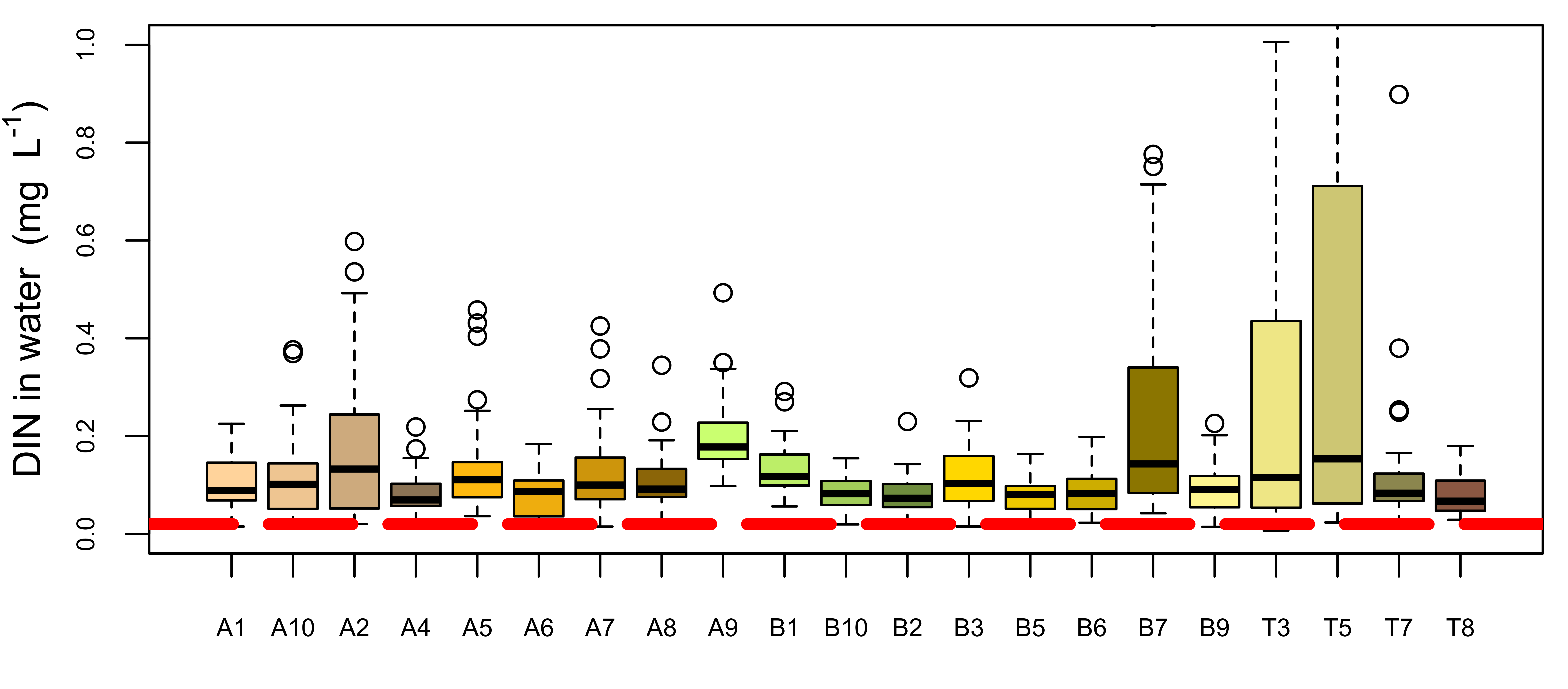

Supplement: Figure S1 — Water column dissolved inorganic nitrogen (DIN) values per pond compared to the experimental N fixation threshold determined by Bradburn et al. [45] . Each box contains per pond DIN values across the growth season. The red dashed line is the DIN threshold (20 μg/L) above which water column N fixation rates decrease substantially. This threshold was determined by field experiments by Bradburn et al. [45] using adjacent water bodies. While DIN concentrations were low in many of the study ponds, DIN levels were consistently maintained above this 20 μg/L threshold suggesting a water column N control on N fixation within the study ponds. (TIFF) [file pone.0095757.s001.tiff]

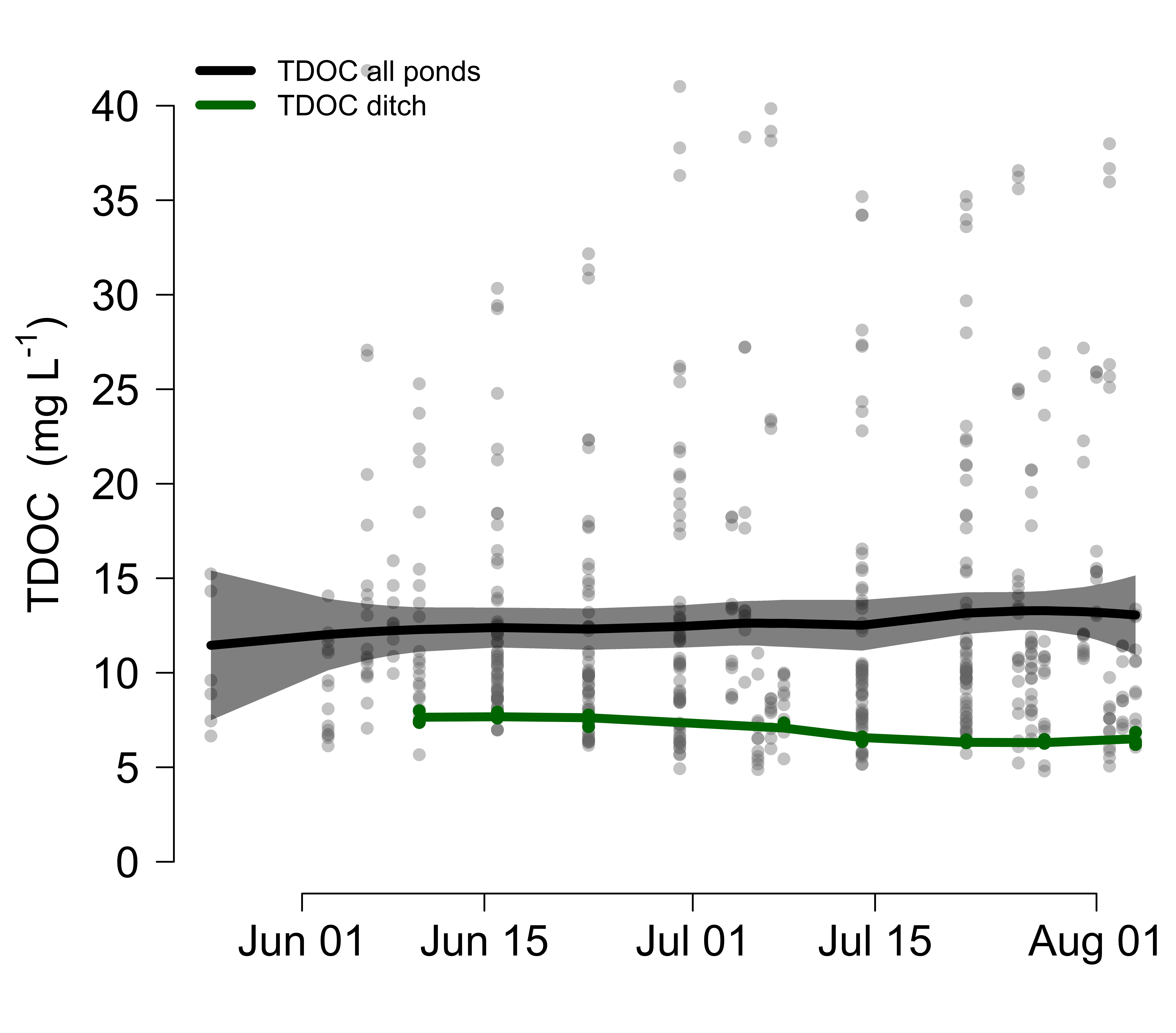

Supplement: Figure S2 — Total dissolved organic carbon (TDOC) concentrations for all ponds across the growth season. TDOC measurements across the growth season remained steady (mean = 13±0.3 mg/L) with no systematic changes across the growth season. The ponds maintained TDOC concentrations above those of the ditch indicating the ditch is not a significant source of TDOC to the ponds. (TIFF) [file pone.0095757.s002.tiff]
