# Supplementary material for: Nitrogen Limitation of Pond Ecosystems on the Plains of Eastern Colorado
Source: PLoS One. 2014 May 13;9(5):e95757. doi: 10.1371/journal.pone.0095757 (PMC4019484; doi:10.1371/journal.pone.0095757)
Supplement: Table S1 — Conductivity, pH, temperature, and dissolved oxygen measurements throughout the 2011 growing season. These are measurements taken in triplicate within ponds across the 2011 growing season: Conductivity (C1,C2,C3), pH (pH1,pH2,pH3), Temperature (T1,T2,T3), and Dissolved Oxygen (DO1,DO2,DO3). (PDF) [file pone.0095757.s003.pdf]

Table S1. Conductivity, pH, temperature, and dissolved oxygen measurements throughout the 2011 growing season.

Site	Date	C1 (μS)	C2 (μS)	C3 (μS)	pH1	pH2	pH3	T1 ($^{\circ}$C)	T2 ($^{\circ}$C)	T3 ($^{\circ}$C)	DO1 (ppm)	DO2 (ppm)	DO3 (ppm)
B9	06/08/11	1159	1194	1142	8.82	7.87	8.87	21.2	20.5	20.2	5.39	4.17	7.57
T5	05/25/11	984	1115	1119	9.01	8.56	8.33	14.3	12.4	12.1	15.26	15.73	13.28
T7	05/25/11	1307	1555	1400	7.2	8.05	8.08	19	24.6	17.4	2.1	6	9.08
B1	06/03/11	1101	1096	1081	9.04	8.87	8.65	19.3	18.8	17.7	13.27	14.72	8.45
B2	06/03/11	1310	1289	1281	8.14	8.22	8.22	20.5	19.2	19	7.1	7.28	7.27
B3	06/03/11	840	877	889	9.65	9.73	9.89	22.8	20.4	21.1	16.28	16.07	17.08
B6	06/03/11	1170	1161	1157	8.85	8.72	8.75	16.3	15.4	15.9	8.75	7.81	6.71
B7	06/03/11	999	985	988	10.11	9.88	10.36	23.5	24.1	25	18	16.33	18
T8	06/04/11	1366	1376	1368	8.71	8.42	8.32	19.3	19.2	20.6	2.84	2.41	5.93
A1	06/06/11	1816	1564	1529	7.6	8.68	8.88	19.3	20.6	21	5.14	7.6	8.68
A2	06/06/11	1208	1156	1182	7.73	8.04	7.7	22.8	21.3	20.2	5.94	8.08	4.57
A4	06/06/11	1776	1779	1849	7.74	8.08	8.21	27.7	26.7	26.3	12.1	7.4	12.31
A5	06/06/11	1638			7.67			28			4.05		
A9	06/06/11	1264	1368	1222	7.86	7.96	7.83	22.6	13.3	14.2	3.53	2.07	3.09
A10	06/06/11	3730	3770	3860	8.3	8.19	7.94	26.6	27.7	26	14.55	8.75	7.6
B5	06/08/11	1274	1283	1284	8.36	8.3	8.13	22.2	22.2	22	9.93	9.65	6.7
B10	06/08/11	1392	1387	1413	8.15	8.41	8.41	28.5	26.2	24.3	10.42	18	11.92
A6	06/10/11	2720	2800	2800	8.14	7.89	7.9	19.4	20.6	20.2	8.68	8.93	4.2
A7	06/10/11	2450	2500	2490	7.82	7.93	8.04	17.7	17.4	16.7	4.61	3.56	2.65
A8	06/10/11	2950	3040	2980	8.15	8.07	8.19	18.1	16.4	17.6	10.37	5.92	11.19
T3	06/10/11	1069	1128		9.15	9.14	9.15	20.2	19.8	20.8	14.51	13.32	14.4
T5	06/10/11	1212	1088	1106	8.71	9.02	9.97	27.5	24.3	22.7	9.49	9.38	18
T7	06/10/11	1249	1498	1374	8.19	7.48	8.12	22.5	24.6	24.1	1.91	4.19	6.93
T8	06/10/11	1321	1349	1322	8.61	8.67	8.47	22.6	23.5	22.1	9.95	10.32	10.67
B7	06/16/11	530	511	510	8.56	8.66	9.16	20.1	20.4	20.5	9.58	4.54	5.02
B3	06/16/11	555	556	554	9.22	9.35	9.37	22.1	22	22.3	9.81	10.54	10.35
B1	06/16/11	1031	1065	1050	9.07	9.04	9.03	21	20.8	20.6	8.66	6.76	6.99
B2	06/16/11	1328	1329	1331	8.19	7.98	8.05	20.3	20.9	20.5	3.28	2.73	3.6
B6	06/16/11	1095	1106	1119	9.02	9.03	9.06	22.5	22.3	27	9.86	9.54	8.57
B9	06/16/11	1179	1184	1182	8.88	8.68	8.61	21.6	21.8	21.5	4.69	5.34	5.92
B5	06/16/11	1277	1281	1270	8.6	8.61	8.58	22.1	22.5	22.8	10.43	10.41	11.02
B10	06/16/11	1388	1384	1383	8.33	8.64	8.66	23.1	23.4	22.8	13.68	15.22	14.45
A1	06/16/11	1532	1538	1528	8.52	8.82	8.45	22.3	22.5	23.1	3.71	7.05	6.98
A2	06/16/11	1234	1265	1256	7.55	7.53	7.57	22.9	22.3	22.7	1.84	4.79	2.19
A7	06/16/11	1438	1475	1460	7.92	8.1	8.02	21.5	21.9	21.6	4.88	6.11	5.3
A10	06/16/11	4370	4280	4290	8.34	8.36	8.5	24.8	23.2	23.1	7.38	7.33	7.5
A8	06/16/11	3070	3100	3210	8.18	8.36	8.34	22.3	23.6	21.6	9.05	12.63	10.11
A9	06/16/11	1332	1308	1343	7.75	7.56	7.63	18.8	19.2	18	3.84	4.54	5.29
A4	06/16/11	1784	1751	1795	8.53	8.29	8.23	23.8	25.1	24	11.97	11.79	13.95
A5	06/16/11	1639	1620	1583	7.71	7.83	7.82	23.9	24.6	23.2	9.98	15.18	4.06
A6	06/16/11	2700	2830	2720	8.05	7.95	7.91	22.9	25.2	25.2	10.05	9.58	9.72
T5	06/16/11	1087	1093	1136	9.14	9.27	8.74	25.7	25.6	24.1	6.78	9.03	7.64
T7	06/16/11	1336	1264	1319	7.71	8.48	8.46	28.7	23.5	23.7	15.37	11.17	8.23
T8	06/16/11	1264	1310	1282	9.19	9.2	9.41	24	22.9	23.1	13.02	12.92	12.94
T3	06/16/11	1089	1111	1094	9.47	9.45	9.44	23.1	22.7	22.6	14.76	15.27	15.42
B6	07/03/11	1205	1229	1241	8.88	8.3	8.83	25.1	24.4	24.8	3.15	1.63	3.93
B5	07/04/11	1322	1336	1346	9.15	9.29	9.32	30.5	30.2	29.8	13.17	18	18
B6	07/04/11	1144	1211	1220	9.44	9.54	9.59	26.9	25.8	25.5	14.14	12.45	11.4
B9	07/04/11	1372	1434	1380	8.82	8.84	9.66	33.3	31.7	31.6	18	18	18
B10	07/04/11	1511	1586	1571	10.31	10.44	10.31	28.9	28.6	28.2	18	18	18
A1	07/05/11	1728	1853	1845	7.79	7.83	7.94	33	27.1	26.5	9.08	4.53	1.93
A2	07/05/11	1291	1390	1375	8.13	8.15	8.03	25.7	25	24.9	2.19	2.67	2.33
A7	07/05/11	2220	2250	2300	8.33	8.35	8.3	24.8	24.7	24.7	7.53	7.13	7.12
A10	07/05/11	4070	4660	4720	8.72	8.16	7.86	28.4	27.5	27.7	1.94	4.05	1.04
B1	07/06/11	1560	1284	1279	8.04	8.83	9.15	28.6	26.1	26.1	3.25	3.62	9.47
B2	07/06/11	1328	1369	1335	9.15	9.17	9.36	30.4	29.5	29.2	18	18	18
B3	07/06/11	455	484	470	9.83	10.19	10.14	23.8	23	22.6	15.51	16.53	16.14
B7	07/06/11	496	492	495	8.46	8.64	8.51	22.9	23.7	21.7	7.33	8.37	5.27
A4	07/07/11	1943	1957	1961	7.82	7.85	7.82	28.7	28.5	28.3	2.72	3.11	2.55
A5	07/07/11	1850	2110	1879	8.85	7.87	8.14	30.1	29	28.9	1.15	4.27	2.17
A6	07/07/11	2720	2850	2750	8.45	8.36	8.55	29.4	29.5	29.2	10.04	9.21	6.3
A8	07/07/11	3130	3140	3260	8.69	8.85	8.65	23.3	23	22.9	1.29	1.74	0.58
A9	07/07/11	1418	1445	1443	8.62	8.05	7.98	12.2	15.5	11.4	2.13	2.35	1.61
T3	07/08/11	1049	1101	1136	9.88	10	9.86	28.5	28.9	28.1	13.7	18	12.59
T5	07/08/11	1059	1118	959	8.56	8.54	8.46	27.9	27.9	27.3	2.56	4.21	2.59
T7	07/08/11	1233	1239	1292	7.74	7.69	7.53	19.5	19.3	19.7	0.35	0.24	0.27
T8	07/08/11	1345	1384	1353	8.42	8.4	8.6	20	20.1	18.2	6.7	7.5	4.65
B5	07/31/11	1243	1293	1311	8.86	8.74	8.7	29.1	28.5	28.8	12.8	11.85	11.63
B9	07/31/11	1671	1658	1690	8.83	8.86	8.61	28.2	29.7	29.1	1.76	2.29	1.31
B10	07/31/11	1309	1334	1381	9.07	9.77	9.8	31.8	31.5	30.8	18	18	18
A1	08/01/11	1746	1752	1763	7.69	7.83	7.89	26.1	23.2	24.7	2.24	1.92	4.26
A2	08/01/11	1368	1395	1395	8.1	8.13	8.2	24.4	24.6	24.5	5.18	4.48	5.13
A7	08/01/11	1922	1930	1946	8.65	8.68	8.66	27.8	26.4	27.9	7.91	9.64	9.63
A10	08/01/11	3510	3600	3570	8.59	8.6	8.67	30.8	30.3	30.7	12.8	13.22	12.93
A4	08/02/11	1769	1948	1982	7.88	8.71	8.59	29.7	29.7	30	14.11	18	15.11
A5	08/02/11	2280	2190	2230	7.49	7.7	7.53	28.4	28.2	28.3	5.11	7.12	6.88
A6	08/02/11	2570	2630	2560	7.72	7.7	8.07	29	29.4	29.2	0.75	0.74	1.32
A8	08/02/11	3380	3380	3360	8.32	8.29	8.37	24.2	23.8	24	3.55	3.54	2.25
A9	08/02/11	1468	1481	1487	7.35	7.38	7.33	17.1	16.8	16.4	0.9	1.03	0.78
B1	08/03/11	1056	1107	1122	8.66	8.43	8.26	26.5	26.5	26.4	14.77	9.33	8.46
B2	08/03/11	1270	1270	1265	10.29	10	10.17	31.1	32.3	31.3	18	18	18
B3	08/03/11	725	532	614	8.71	8.76	9.02	23.8	23.8	23.8	5.74	11.78	9.53
B7	08/03/11	1133	1082	1093	7.81	7.87	7.85	23.2	23.3	23.3	4.51	8.55	4.56
T3	08/04/11	1271	1284	1306	9.35	9.58	9.55	26.1	27.2	26.6	12.21	18	18
T5	08/04/11	1143	1181	1151	7.79	7.58	7.68	24.3	24	25.9	0.61	0.38	1.28
T7	08/04/11	1235	1232	1235	7.3	7.35	7.36	19	19.1	19.3	0.26	0.5	0.4

These are measurements taken in triplicate within ponds across the 2011 growing season: Conductivity (C1,C2,C3), pH (pH1,pH2,pH3), Temperature (T1,T2,T3), and Dissolved Oxygen (DO1,DO2,DO3).
