# Supplementary material for: Nitrogen Limitation of Pond Ecosystems on the Plains of Eastern Colorado
Source: PLoS One. 2014 May 13;9(5):e95757. doi: 10.1371/journal.pone.0095757 (PMC4019484; doi:10.1371/journal.pone.0095757)
Supplement: Table S2 — Water Chemistry and Seston Data for Waters Used in 2012 Experiments. (PDF) [file pone.0095757.s004.pdf]

Table S2. Water Chemistry and Seston Data for Waters Used in 2012 Experiments.

pond name	Nitrate-N mg/L	Ammonium-N mg/L	DIN mg/L	TDP mg/L	DIN:TDP mass	Seston N:P molar	N fixation rate μ mol N/L/day	Nutrient Limitation
A1	0.028	0.085	0.113	0.0172	6.6	6.6	0.054	P
A2	0.004	0.046	0.050	0.0582	0.9	7.9	0.054	N
A4	0.006	0.011	0.017	0.0398	0.4	12.1	0.057	Nseq
A5	0.011	0.070	0.081	0.0124	6.5	3.0	0.057	NPdual
A6	0.006	0.027	0.034	0.2321	0.1	9.1	0.050	N
A7	0.021	1.460	1.481	0.0508	29.2	3.5	0.052	–
A8	0.009	0.045	0.053	0.7180	0.1	13.4	0.066	N
A9	0.009	0.058	0.067	0.0579	1.2	5.1	0.041	–
A10	0.007	0.004	0.011	0.0279	0.4	9.4	0.048	N
B1	0.012	1.848	1.860	0.1762	10.6	8.5	0.058	–
B2	0.006	0.055	0.061	0.0206	3.0	3.1	0.046	NPdual
B5	0.007	0.075	0.082	0.0702	1.2	36.7	8.896	–
B6	0.024	0.417	0.441	0.6165	0.7	4.4	0.063	N
B7	0.005	0.050	0.055	0.0299	1.8	6.4	0.046	NPco
DITCH	0.001	0.007	0.008	0.217336	0.0	12.2	0.108	N
T3	0.001	0.059	0.060	0.0340	1.8	2.8	0.178	Nseq
T5	0.006	0.057	0.064	0.1762	0.4	3.8	0.048	N
T7	0.007	0.061	0.067	0.0204	3.3	13.5	0.063	–
